# A Quantitative Comparison of ^31^P Magnetic Resonance Spectroscopy RF Coil Sensitivity and SNR between 7T and 10.5T Human MRI Scanners Using a Loop-Dipole ^31^P-^1^H Probe

**DOI:** 10.3390/s24175793

**Published:** 2024-09-06

**Authors:** Xin Li, Xiao-Hong Zhu, Wei Chen

**Affiliations:** Center for Magnetic Resonance Research (CMRR), Department of Radiology, University of Minnesota, Minneapolis, MN 55455, USA; li002316@umn.edu (X.L.); zhuxx022@umn.edu (X.-H.Z.)

**Keywords:** magnetic resonance imaging (MRI), magnetic resonance spectroscopy imaging (MRSI), ultrahigh field, 7T, 10.5T, phosphorus-31 (^31^P) MRSI, RF coil

## Abstract

In vivo phosphorus-31 (^31^P) magnetic resonance spectroscopy (MRS) imaging (MRSI) is an important non-invasive imaging tool for studying cerebral energy metabolism, intracellular nicotinamide adenine dinucleotide (NAD) and redox ratio, and mitochondrial function. However, it is challenging to achieve high signal-to-noise ratio (SNR) ^31^P MRS/MRSI results owing to low phosphorus metabolites concentration and low phosphorous gyromagnetic ratio (γ). Many works have demonstrated that ultrahigh field (UHF) could significantly improve the ^31^P-MRS SNR. However, there is a lack of studies of the ^31^P MRSI SNR in the 10.5 Tesla (T) human scanner. In this study, we designed and constructed a novel ^31^P-^1^H dual-frequency loop-dipole probe that can operate at both 7T and 10.5T for a quantitative comparison of ^31^P MRSI SNR between the two magnetic fields, taking into account the RF coil B_1_ fields (RF coil receive and transmit fields) and relaxation times. We found that the SNR of the ^31^P MRS signal is 1.5 times higher at 10.5T as compared to 7T, and the power dependence of SNR on magnetic field strength (B_0_) is 1.9.

## 1. Introduction

*In vivo* phosphorus-31 (^31^P) magnetic resonance spectroscopy (MRS) imaging (MRSI) detects various phosphorus metabolites involving adenosine triphosphate (ATP) energy and phospholipid metabolisms, as well as the nicotinamide adenine dinucleotide (NAD) and NAD redox ratio in human brain [1,2,3,4,5]. The abnormality observed in the ^31^P MRS studies reflects brain pathology and diseases, such as ischemia, seizure, epilepsy and Alzheimer’s disease [6,7,8,9]. However, it is still challenging to achieve high spatiotemporal resolution for investigating regional phosphorus metabolic content and abnormality owing to the very low cerebral concentration of phosphorus metabolites and the low ^31^P gyromagnetic ratio (γ), and thus, low sensitivity or signal-to-noise ratio (SNR), as compared to proton (^1^H). Previous studies have shown that increasing magnetic field strength (*B*_0_) for 7T and above can largely improve the ^31^P MRS SNR and spectral resolution [10,11,12,13,14,15,16]. The 10.5T whole-body imaging system is among the highest magnetic field strength MRI scanners for human whole-body imaging in the world. A number of in vivo proton (^1^H)-based MRI studies at 10.5T have demonstrated significantly improved human brain and body imaging results compared to 7T [17,18,19]. However, the field-dependent ^31^P MRS/MRI SNR improvement from 7T to 10.5T remains unknown, and there is a lack of experimental studies that quantitatively compare the ^31^P SNR improvement between 10.5T and 7T human MRI scanner, as differences in radio-frequency (RF) coil designs for both magnetic fields could affect the comparisons and outcomes [20]. In addition, there is a lack of field-dependent X-nuclear (e.g., ^31^P, ^17^O and ^2^H) MRS SNR quantification studies that quantitatively consider the RF coil sensitivity profiles. Traditional studies for field-dependent SNR quantification of X-nuclei SNR involving small RF coil sizes or small animals [10,11,21,22] have only considered the RF coil quality factor (Q factor) in loaded conditions. For larger RF coil sizes or higher operating frequencies such as ^31^P at 10.5T (operating frequency = 180.5 MHz), the RF coil Q factor may not be sufficient to account for the factor on SNR, and the RF coil sensitivity profile needs to be considered [23,24,25].

The combination of dipole and monopole with a loop resonator has become a popular choice of dual- or even triple-frequency tuned RF coils used in X-nuclear and proton imaging in ultrahigh field MRI [26,27,28]. In an overlapped dipole and loop design, the magnetic fluxes generated by the dipole coil and loop coil are orthogonal, resulting in intrinsic decoupling between the two coils [27]. In addition, the Poynting vector of the radiative dipole is pointed toward the phantom and allows a strong far-field regime or deeper penetration of B_1_ fields (transmit and receive magnetic fields) in imaging phantom at ultrahigh field, while the near-field regime is dominant and the B_1_ penetration is shallower for the loop resonator [29]. Thus, the dipole coil is typically placed on the outer layer and the loop coil is placed on the inner layer of the multi-layer RF coil designs to optimize the performance for both coil types in multi-frequency MRI/MRS applications.

In this work, we designed and applied the same ^31^P-^1^H dual-frequency loop-dipole RF coil consisting of a ^31^P surface loop coil for ^31^P MRSI and a ^1^H dipole coil for ^1^H-based structural imaging and B_0_ shimming at both 7T and 10.5T. The coil shows stable match and tuning for both ^31^P and ^1^H channels and good decoupling between the ^1^H and ^31^P channels at both 7T and 10.5T human scanners. It is used to perform ^31^P MRSI at both fields to quantitatively compare the SNR difference between the two fields in consideration of the multiple factors of *B*_0_, RF coil B_1_ fields, the longitudinal relaxation time (T_1_) and ^31^P spectral linewidth at half peak height (∆*v*).

## 2. Materials and Methods

### 2.1. RF Coil Construction

**Figure 1:**
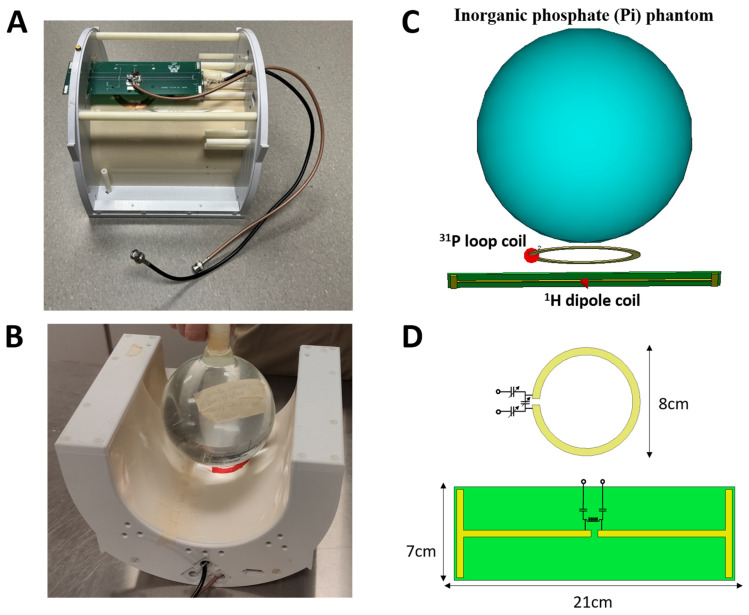
The phosphorus-31 (^31^P)–proton (^1^H) loop-dipole probe was placed inside a coil former below an inorganic phosphate (Pi) water phantom. (**A**) The ^31^P-^1^H loop-dipole probe, (**B**) the actual imaging setup for performing ^1^H MRI and ^31^P MRSI of the Pi phantom in the 7T and 10.5T human whole-body MRI scanners, (**C**) the graphical demonstration and (**D**) the RF coil dimensions and schematics of match/tuning networks.

**Figure 2:**
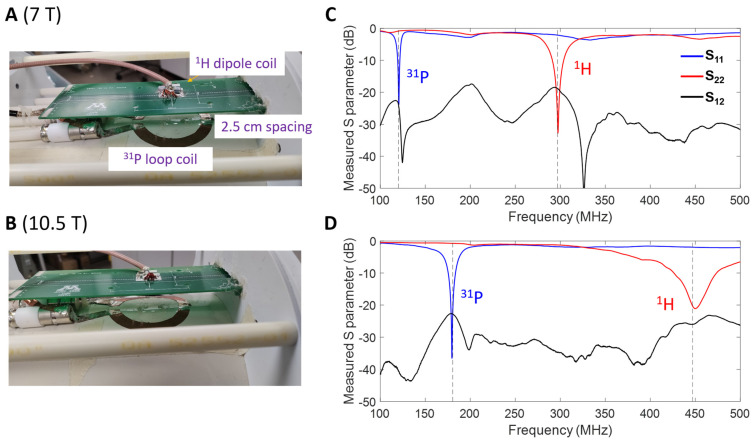
Prototype of a ^31^P-^1^H loop-dipole probe consisting of a PCB-printed ^1^H dipole coil on the top of an 8 cm diameter ^31^P surface loop coil, with a 2 cm gap between the dipole and loop coils. This loop-dipole coil can be tuned and matched to the operating frequencies of 120.3 MHz for ^31^P and 297 MHz for ^1^H at 7T (**A**), and 180.5 MHz for ^31^P and 447 MHz for ^1^H at 10.5T (**B**). (**C**) In loaded condition, the network analyzer (Rohde & Schwarz, Munich, Germnay) measurements show great S_11_ and S_22_ (reflection coefficients) for the 7T ^31^P loop and ^1^H dipole, respectively, with S_12_ (coupling coefficient) between the ^31^P loop and dipole coils being less than −20 dB at 120.3 MHz. (**D**) In loaded condition, the measured reflection coefficients for 10.5T ^31^P loop (S_11_) and ^1^H dipole coil (S_22_). The maximum coil coupling (S_12_) between the ^31^P loop and dipole coil is less than −20 dB at 180.5 MHz.

The ^31^P MRSI experiments were performed using an inorganic phosphate (Pi) water phantom on both 7T and 10.5T magnetic field strength human scanners (SIEMENS, Munich, Germany), and a ^31^P-^1^H dual-frequency loop-dipole probe was used as a RF coil, as shown in Figure 1. The dipole and loop coils are mounted on a low dielectric loss polycarbonate RF coil former. The same coil former setup (Figure 1A,B) is used for ^1^H imaging, B_0_ shimming and ^31^P MRSI at both magnetic fields. We designed and constructed the ^31^P-^1^H dual-frequency loop-dipole probe with passive decoupling, which could be operated at both 7T and 10.5T operation frequencies. The dipole and loop coil dimensions and the match/tuning networks are shown in Figure 1C,D. The ^1^H dipole coil is milled on a PCB board (21 cm length). The match/tuning circuit for the dipole coil consists of a shunt inductor and two in-series capacitors, as shown in Figure 1D. Two match/tuning circuits with different inductor and capacitor values were used for the matching and tuning of the dipole coil at 297 MHz (7T) and 447 MHz (10.5T) ^1^H operation frequencies, respectively. The ^31^P loop coil is built using a 1.4 mil thickness copper foil shielding tape, forming an 8 cm outer diameter circle. The L-type match/tuning network consisting of three trimmer capacitors is used for the ^31^P loop coil. This match/tuning network allows for the adjustment of the tuning frequency of the ^31^P loop coil from 120.3 MHz (7T) to 180.5 MHz (10.5T). The ^1^H dipole coil is located 2.5 cm away from the ^31^P loop coil plane. This 2.5 cm gap between the dipole and loop coil allows for sufficient decoupling between the two coils at both ^31^P and ^1^H operating frequencies. A closer distance between the loop and dipole coil can result in more coupling between the two coils. The RF coil Q factor was measured using −7 dB from the 0 dB baseline of the S_11_ (scattering parameter) plot, as demonstrated in Figure 2C,D, based on the Q factor calculation method described in the literature [30].

### 2.2. ^31^P MRSI Experiment

Figure 1C shows the setup of the loop-dipole coil loaded with the Pi phantom. The Pi phantom contains 100 mM NaH_2_PO_4_ and 0.05 mM Gadolinium (Gd for shortening the Pi T_1_ value) and has a total volume of 2 L. The loading effect of the Pi phantom is similar to a human head. B_0_ shimming was performed over an 8 cm cube volume at the bottom of the phantom near the RF coil plane. Three-dimensional (3D) ^31^P chemical shift images (CSI) [31] with different RF excitation pulse voltages (square pulse shape) were acquired with a field of view (FOV) of 15 × 15 × 15 cm and matrix size of 9 × 9 × 7, covering the entire phantom. At both magnetic fields, we used the same CSI acquisition parameters: repetition time (TR) = 1500 ms (fully relaxed condition), echo time (TE) = 0.5 ms, excitation hard pulse length = 500 μs and spectral width = 5000 Hz. With the Gd solved in the phantom, the T_1_ of Pi is approximately 300 ms at 7T and 220 ms at 10.5T; thus, a 1500 ms TR is sufficient to achieve full relaxation conditions at both fields. For data post processing, the Pi FID (Free Inductive Decay) data from each CSI voxel was converted to a spectrum using FFT, with a 10 Hz exponential apodization and zero filling with 5000 points. For the spectrum conversion, a 0-degree first-order phase shift is used, and an optimal zero-order phase shift is used to maximize the real part of the Pi spectral peak height. After applying both first-order and zero-order phase corrections, the peak height of the real part of the Pi spectrum is used as the measured Pi signal.

### 2.3. SNR Quantification

For 3D Fourier-series Window (FSW) CSI [31,32], the measured ^31^P MRS signal (Pi spectral peak) in a given voxel [33] is as follows:

(1) $$S=M_{0}\cdot B_{1}^{-}\cdot sin(\alpha ),$$

where S is the measured signal of ^31^P MRS, $M_{0}$ is the equilibrium magnetization along the *z*-axis, with its magnitude depending on the magnetic field strength [34]. $B_{1}^{-}$ is the RF coil receive field. The RF pulse flip angle α is given by

(2) $$\alpha =\gamma \cdot \tau \cdot {|B}_{1}^{+}|\cdot V,$$

where $B_{1}^{+}$ is the RF coil transmit field normalized by excitation pulse voltage, in units of *T*/*volt*, γ is the gyromagnetic ratio, τ is the time duration of RF excitation pulse and *V* is the RF excitation pulse voltage.

The 90-degree flip angle ^31^P signal ($S_{90{}^{\circ}}$) and the corresponding $B_{1}^{+}$ fields are quantified for all ^31^P 3D CSI voxels. For each CSI voxel, the measured ^31^P spectral peak heights with different RF excitation voltages were fitted to a sine function, where the excitation voltages are used as the dependent variable and the spectral peak heights are used as the independent variable. The spectral peak heights determined by the regression of the sine wave function at the flip angle of 90° represent the ultimate (or largest) ^31^P signal ($S_{90{}^{\circ}}$) that could be achieved in a particular voxel. At $S_{90{}^{\circ}}$, sin(α) equals to one, and the corresponding RF excitation voltage is proportional to $1/B_{1}^{+}$, and can determine the $B_{1}^{+}$ value according to Equation (2). The quantification of $S_{90{}^{\circ}}$ and $B_{1}^{+}$ fields is described in detail in the literature [35]. We selected 10 voxels from similar locations near the center of the coil’s sensitive region to calculate the mean ^31^P signal ($S_{90{}^{\circ}}$) for 7T and 10.5T, respectivly. To quantify the spectral noise level, we acquired the ^31^P CSI with 0-*volt* RF pulse voltage. The standard deviation from the inner band of the noise spectra was calculated to obtain the spectral noise ($\sigma_{noise}$) for both 7T and 10.5T.

Under fully relaxed acquisition conditions with a 90-degree flip angle (α = 90°), and by dividing the ^31^P signal (Equation (1)) with spectral noise level ($\sigma_{noise}$), the ^31^P SNR can be calculated [10,11] and be described by

(3) $${SNR\propto B}_{0}^{\beta }\cdot \sqrt{Q_{loaded}}\cdot \sqrt{\frac{1}{T_{1}\cdot ∆v}}\;,$$

where $B_{0}^{\beta }$ is the magnetic field-dependent term with a power factor of β, $Q_{loaded}$ is the RF coil quality factor in the loaded condition. The SNR ratio between 10.5T and 7T is

(4) $$\frac{{SNR}_{\;31P\;10.5T}}{{SNR}_{\;31P\;7T}}={\left(10.5/7\right)}^{\beta }\cdot \sqrt{\frac{Q_{loaded\;10.5T\;}}{Q_{loaded\;7T}}}\cdot \sqrt{\frac{T_{1\;7T\;}{\cdot \;∆v}_{\;7T}}{{T_{1\;10.5T}\;\cdot \;∆v}_{\;10.5T}}}\;.$$

The $Q_{loaded}$ can be related to the RF coil sensitivity by

(5) $$\sqrt{Q_{loaded}}=B_{1}^{-}\cdot \sqrt{\omega }\;,$$

where ω is the Larmor frequency. A modified SNR equation is

(6) $$SNR\propto B_{0}^{\beta }\cdot B_{1}^{-}\cdot \sqrt{\omega }\cdot \sqrt{\frac{1}{T_{1}∆v}}\;.$$

Due to the principle of reciprocity and weak RF wave behavior at a low RF coil operating frequency [24,32], the $B_{1}^{-}$ field in *T*/*volt* at a relatively lower frequency (180.5 MHz or below) for a single-channel surface loop is approximately equal to $B_{1}^{+}$ field. Thus, Equation (6) becomes

(7) $${SNR\propto B}_{0}^{\beta }\cdot B_{1}^{+}\cdot \sqrt{\omega }\cdot \sqrt{\frac{1}{T_{1}∆v}}\;.$$

The SNR ratio between 10.5T and 7T ^31^P MRSI is as follows:

(8) $$\frac{{SNR\;}_{31P\;10.5T}}{{SNR}_{\;31P\;7T}}={\left(10.5/7\right)}^{\beta +1/2}\cdot \frac{\;{B_{1}^{+}}_{31P\;10.5T}}{{B_{1}^{+}}_{31P\;7T}\;}\cdot \sqrt{\frac{T_{1\;7T}{\;\cdot \;∆v}_{\;7T}}{{T_{1\;10.5T\;}\cdot \;∆v}_{\;10.5T}}}\;.$$

## 3. Results

### 3.1. RF Coil Performance

The ^31^P loop coil and ^1^H dipole coil under similar conditions or setups are used for the ^31^P MRSI and ^1^H MRI studies at 7T (Figure 2A) and 10.5T (Figure 2B). As shown in Figure 2C,D, the ^31^P loop coil and ^1^H dipole coil were tuned and matched to S_11_ (reflection coefficient) of less than −20 dB for ^31^P and ^1^H, respectively, at both 7T and 10.5T operating frequencies in the loaded conditions. As our study mainly focuses on ^31^P SNR quantification, coil coupling at ^31^P operating frequencies is more important than coil coupling at the ^1^H operating frequencies at both magnetic fields. The S_12_ (coupling coefficient) between the ^31^P loop and ^1^H dipole coils was less than −20 dB at the ^31^P operating frequencies and close to −20 dB at the ^1^H operating frequencies, indicating excellent decoupling between the ^31^P and ^1^H coils and providing reliable imaging performance for the ^31^P CSI acquisition at both magnetic fields.

### 3.2. ^1^H Imaging and ^31^P MRSI Results

**Figure 3:**
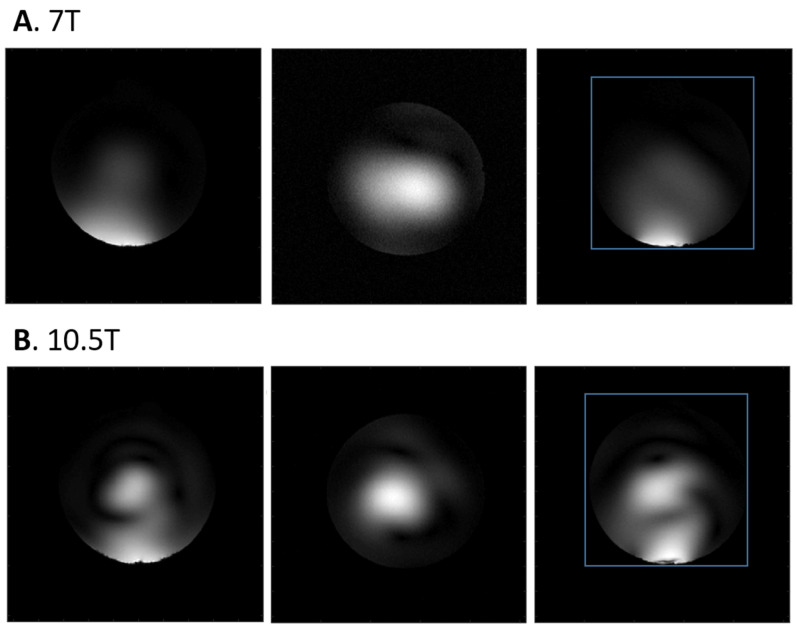
The ^1^H MRI localizer images at (**A**) 7T and (**B**) 10.5T, respectively, in the sagittal, coronal and transversal orientations. The RF coil is placed beneath the Pi Phantom. The blue box shows the 15 cm FOV selection for ^31^P CSI. Stronger wave effects can be observed in the 10.5T localizer images.

Figure 3 demonstrates the ^1^H-based MRI localizer at both 7T and 10.5T. Sagittal, coronal and axial orientation images are shown in Figure 3A and Figure 3B for 7T and 10.5T, respectively. Stronger RF wave behaviors can be observed in the sagittal and axial orientations of the ^1^H localizer images at 10.5T compared to 7T.

**Figure 4:**
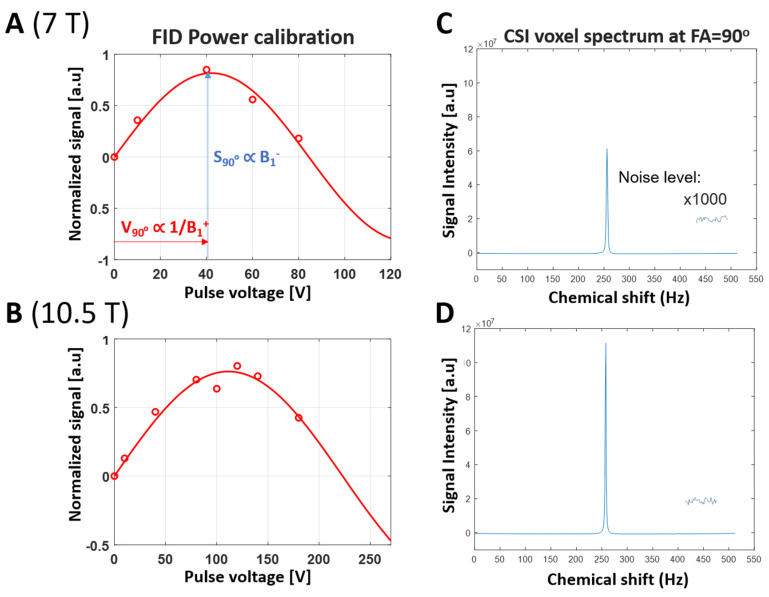
Illustration for determining the maximum Pi signal ($S_{90{}^{\circ}}$) value for a selected CSI voxel. (**A**) Measured ^31^P spectral peak signal and hard pulse voltage fitting for a representative CSI voxel with the largest intensity ($S_{90{}^{\circ}}$) for 7T. For each voxel, the spectral peak heights of multiple Pi spectra acquired with varied RF pulse voltages were fitted with a sine function to determine the $S_{90{}^{\circ}}$ at the 90-degree nominal flip angle (FA). (**B**) ^31^P Spectral peak signal and pulse voltage fitting for a representative CSI voxel with the largest intensity ($S_{90{}^{\circ}}$) at 10.5T. (**C**,**D**) The ^31^P spectrum of a representative CSI voxel near the 90-degree nominal FA, thus, with the largest intensity for 7T and 10.5T, respectively. The spectral noise levels are zoomed in 1000 times and show similar levels for both 7T and 10.5T.

**Figure 5:**
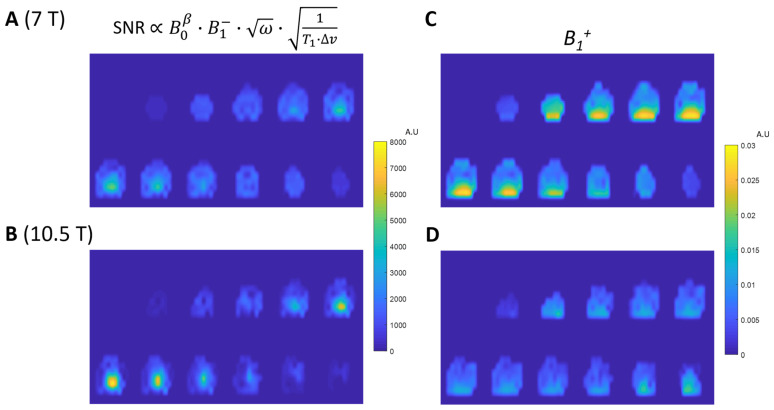
The SNR maps of ^31^P MRSI at 90-degree pulse voltage under fully relaxed conditions for (**A**) 7T and (**B**) 10.5T, where the SNR is proportional to the magnetic field strength dependence term ($B_{0}^{\beta }$) and the *B*_1_*^−^* field (RF coil receive field). (**C**) The *B*_1_*^+^* field (RF coil transmit field) maps normalized by excitation pulse voltage for 7T ^31^P (operating frequency = 120.3 MHz). (**D**) *B*_1_*^+^* field maps for 10.5T ^31^P (operating frequency = 180.5 MHz).

We determined the $B_{1}^{+}$ values for all CSI voxels based on the fitted voltage map for 90-degree flip angles for all CSI voxels. This is achieved by applying the “sine” function fitting for all 3D ^31^P CSI voxels to determine the 3D distribution of $S_{90{}^{\circ}}$ and corresponding 90-degree flip angle voltage values for both 7T and 10.5T. The fitting method is also described in detail in Section 2.3. Figure 4 illustrates the quantification method using sine function fitting to determine $S_{90{}^{\circ}}$ and the corresponding 90-degree excitation voltage for a representative CSI voxel at 7T (Figure 4A) and 10.5T (Figure 4B). Figure 4C,D show the ^31^P spectra of two representative CSI voxels near the 90-degree nominal FA, and thus with the highest intensity for 7T and 10.5T, respectively. Figure 4C,D also show the spectral noise level zoomed in 1000 times, indicating similar noise levels for both 7T and 10.5T, and thus, a higher SNR at 10.5T. Figure 5A,B show the SNR maps ($S_{90{}^{\circ}}$ divided by spectral noise level) for 7T and 10.5T, respectively. It shows significant improvement in the SNR maps at 10.5T compared to 7T. On the other hand, the $B_{1}^{+}$ maps at 10.5T (Figure 5D) show a significant decrease compared to the $B_{1}^{+}$ maps at 7T (Figure 5C). The decrease in $B_{1}^{+}$ at 10.5T is also reflected by the reduction in loaded RF coil Q factor ($Q_{loaded}$) at 10.5T compared to 7T. In addition, a slightly higher noise level ($\sigma_{noise}$) is observed in 10.5T compared to 7T, due to increased sample and RF coil loss at higher magnetic fields. The $Q_{loaded}$ and $\sigma_{noise}$ values for both magnetic fields are reported in Table 1.

### 3.3. SNR Quantification and Comparison between 7T and 10.5T

**Figure 6:**
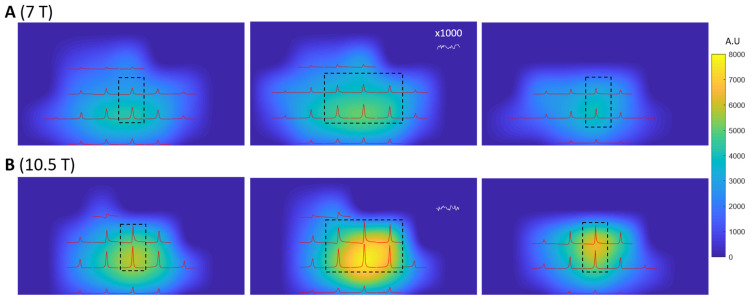
Three representative transversal slices of ^31^P SNR maps of Pi acquired at 7T (**A**) and 10.5T (**B**), overlaid with 2D CSI slice (extracted from 3D CSI data) displayed with same vertical scale, acquired at 90-degree flip angle based on the global FID power calibration. The spectral noise level from one peripheral CSI voxel for 7T and 10.5T was zoomed in ×1000 times along the vertical scale, and both fields show similar spectral noise levels. This figure clearly shows a significant improvement in spectral quality and SNR at 10.5T.

Figure 6 shows the ^31^P SNR maps of three transversal slices at both field strengths. These SNR maps are overlaid with the 3D-CSI ^31^P spectra acquired using the excitation voltage corresponding to the nominal 90-degree flip angle for the global FID signals. Ten voxels (within the dash-line black box in Figure 6) are selected to calculate the average ^31^P SNR for 7T and 10.5T. The spectral noise from a CSI voxel in the peripheral region for 7T and 10.5T is zoomed in 100 times, showing similar noise levels between the two magnetic fields. Table 1 summarizes the parameters of T_1_, loaded RF coil Q factor ($Q_{loaded}$), average SNR, average $B_{1}^{+}$, average spectrum linewidth (∆*v*) for the selected 10 voxels shown in Figure 6, and noise level ($\sigma_{noise}$) acquired and quantified at 0-*volt* RF excitation pulse voltage at 10.5T and 7T, respectively. The ratio of the SNRs measured at 10.5T to 7T is 1.48 ± 0.2. However, the average $B_{1}^{+}$ and $Q_{loaded}$ are lower at 10.5T compared to 7T. Substituting the ratio of average SNR, T_1_, linewidth (Δ*v*) and average $B_{1}^{+}$ into Equation (8), we obtain $1.48={\left(\frac{10.5}{7}\right)}^{\beta }\times \sqrt{\frac{1}{0.73\times 0.95}}\times 0.45\times \sqrt{1.5}$. This yields a magnetic field-dependent power factor (β) of 1.9. However, if use traditional SNR quantification according to Equation (4), we obtain $1.48={\left(\frac{10.5}{7}\right)}^{\beta }\times \sqrt{\frac{1}{0.73\times 0.95}}\times \sqrt{0.54}$. This yields a magnetic field-dependent power factor (β) of 1.3.

## 4. Discussion

We used a novel ^31^P-^1^H dual-frequency loop-dipole RF coil for ^31^P CSI imaging, which also supports ^1^H-based structural imaging and B_0_ shimming, and demonstrated excellent ^31^P MRSI performance at both 7T and 10.5T human scanners. The dipole–loop coil exhibits excellent decoupling between the ^31^P and ^1^H channels and allows for tuning adjustments between 7T and 10.5T for both ^31^P and ^1^H operating frequencies. Using the same imaging setup at both fields, we conducted a quantitative comparison of the coil Q factor, RF coil *B*_1_ fields and ^31^P SNR between 7T and 10.5T. We observed a ^31^P MRSI SNR ratio of 1.48 between 10.5T and 7T human scanners (with a B_0_ ratio of 1.5), indicating an approximately linear relationship with B_0,_ consistent with previous reports [10,11].

Additionally, we considered the RF coil sensitivity profile in the proposed ^31^P MRSI SNR quantification method. As the magnetic field strength increases, the imaging frequency also rises, leading to a stronger RF wave effect [36] and a decrease in RF coil sensitivity [24,37]. The spectral linewidth effect, T_1_, spectral noise and RF coil sensitivity differences at both 7T and 10.5T were used to derive the power factor (β) of the magnetic field-dependent term ($B_{0}^{\beta }$). Under fully relaxed conditions (TR = 1.5 s) for both 7T and 10.5T measurements, and using 90-degree flip angle voltage for excitation, we simplified the SNR calculation equations and derived β = 1.9 from Equation (8) using the parameters listed in Table 1, which is close to the theoretical value of β = $\frac{7}{4}$ based on the reports in the literature [38,39,40] for low operating frequency where the coil loss is dominated. For higher frequency, as a RF coil that can perform close to the optimum for each magnetic field strength, the ultimate possible value of intrinsic SNR is dominated by sample loss and the optimal electromagnetic field distribution inside the sample, and even higher SNRs (β > $\frac{7}{4}$) are achievable [41,42,43]. The magnetic field-dependent power factor based on Equation (8) is higher than the result obtained from Equation (4), which provides β = 1.3, and also higher than previously reported values in [10], where a smaller RF coil (coil diameter = 5 cm) was used, and β was found to be 1.4 in the human brain at 4T and 7T; similarly, another study reported β values of 1.4–1.5 for small coil and small animal [11]. These previous studies did not account for RF coil sensitivity in SNR quantification, although the RF coil $Q_{loaded}$ was considered. The $Q_{loaded\;}$ exhibits similar frequency-dependent behavior as the RF coil sensitivity and decreases with increasing field strength due to the rise in the RF coil operating frequency [23,40]. However, $Q_{loaded\;}$ is only indirectly related to RF coil sensitivity, and Equation (4) may have underestimated the RF coil sensitivity’s influence on the measured ^31^P signal, especially for larger loop coil and higher RF coil operating frequency for human imaging. In addition, it is challenging to obtain accurate $Q_{loaded\;}$ value, as $Q_{loaded\;}$ is usually measured on the benchtop with a network analyzer rather than in the scanner. The $Q_{loaded\;}$ measurement accuracy can be significantly affected by the RF coil’s tuning and matching capabilities, as well as by the proper shielding of electromagnetic fields from the surroundings [30].

A limitation of this study is that we did not use cable traps or baluns for the ^31^P-^1^H dual-frequency loop-dipole coil for the 7T and 10.5T imaging studies. We are concerned that the cable traps or baluns tuned to ^31^P and ^1^H frequencies for 7T imaging will not be applicable for the ^31^P and ^1^H imaging at 10.5T, and could also affect the imaging performance at 10.5T. Thus, we did not use any cable traps or balun for the RF coil when comparing the imaging performance between the two magnetic field strengths. However, magnetic cable traps were used for the coil matchings and tunings in loaded condition on the bench.

## 5. Conclusions

We conducted the first study using a 10.5T human scanner to report an approximately 1.5-fold gain in ^31^P MRS/MRSI SNR compared to a 7T human scanner. Additionally, we also investigated and demonstrated a magnetic field-dependent power factor of 1.9 for ^31^P MRSI SNR through a quantitative comparison of ^31^P MRSI results between 7T and 10.5T human scanners using a ^1^H/^31^P dipole–loop RF coil. Considering the 1.5 times SNR gain and the anticipated improvement in spectral resolution at 10.5T, we expect significant improvements for in vivo ^31^P MRSI for human brain application at 10.5T. The findings from our work with the dual-tuned ^31^P-^1^H dipole–loop will aid in the design of future dipole–loop array coils for human ^31^P MRSI in the 10.5T human scanner. This is particularly promising for challenging applications such as measuring the brain ATP and creatine kinase fluxes and separating and quantifying the reduced and oxidized NAD signals *in vivo*.

## Figures and Tables

**Table 1:** Comparison of 10.5T and 7T ^31^P RF coil and MRS imaging parameters and their ratios.

*B*_0_	T_1_ (ms) (with Gd)	∆*v* (Hz)	Q_loaded_	SNR (10 Voxels Average)	$B_{1}^{+}$ (10 Voxels Average)	$\sigma_{noise}$
10.5T	220	27.6	40.9	5.57 ± 0.89 × 10^3^	10.9 × 10^−3^	1.9 × 10^4^
7T	300	29.0	75.3	3.82 ± 0.69 × 10^3^	24.4 × 10^−3^	1.4 × 10^4^
ratio	0.73	0.95	0.543	1.48 ± 0.24	0.45	1.35

## Data Availability

The data used during the current study are available by the authors on request.
